# *Vanilla planifolia*: Artificial and Insect Pollination, Floral Guides and Volatiles

**DOI:** 10.3390/plants13212977

**Published:** 2024-10-25

**Authors:** Sahar Van Dyk, Williams Barry McGlasson, Mark Williams, Robert Spooner-Hart, Paul Holford

**Affiliations:** School of Science, Western Sydney University, Locked Bag 1797, Penrith 2751, Australiam.williams@westernsydney.edu.au (M.W.); r.spooner-hart@westernsydney.edu.au (R.S.-H.); p.holford@westernsydney.edu.au (P.H.)

**Keywords:** *Tetragonula carbonaria*, *Austroplebeia australis*, *Lucilia cuprina*, pollination guides, melezitose, floral volatiles, extrafloral nectaries

## Abstract

The natural pollinator of the major species of commercially-grown vanilla, *Vanilla planifolia*, is unknown, and the crop requires hand pollination to achieve significant levels of fruit set; however, the traditional technique (using a toothpick) is costly, as it requires skilled personnel. To overcome this problem, two native Australian bees, *Tetragonula carbonaria* and *Austroplebeia australis*, and the blowfly, *Lucilia cuprina*, were trialled as pollinators. Three alternatives to the toothpick method were also trialled. The appearance of vanilla flowers under ultraviolet radiation was examined to determine the presence of cryptic pollination guides, and the chemical composition of nectar from extrafloral nectaries and aroma volatiles from the flowers were characterised. None of the three insects effected pollination due to their small size and behaviour; other insect pollinators need to be identified. The alternative mechanical methods of pollination trialled resulted in fruit set; however, the percentages of fruit set were lower than the traditional toothpick method, and the fruit were of inferior quality. The nectar produced predominantly consisted of sucrose and melezitose. Melezitose is a strong attractant of various ant species, which may explain the concentration of ants around the nectaries and the apparent lack of nectar production in part of this study. The aroma volatiles included monoterpenoids, terpenes, sesquiterpenoids, aromatics, nitrogen-containing compounds and fatty acids, the most abundant being a-pinene and eucalyptol. Illumination of the flowers with UV-A radiation revealed fluorescence from the stamens, the column and the callus, which is located on the labellum. These observations may aid the identification and use of potential pollinators.

## 1. Introduction

Pollination is a key ecological process affecting sustainable agricultural production [1] and refers to the transfer of pollen to the stigma, either by close proximity of the anther (self-pollination) or by biotic and abiotic agents (cross-pollination). Most plants of the genus *Vanilla* (Asparagales: Orchidaceae) have flowers that are self-fertile [2]. Within this genus, three species are grown commercially, *V. tahitensis* J.W. Moore, *V. pompona* Schiede and *V. planifolia* Andrews [3] with the latter being the most important and is referred to as vanilla throughout this study. Plants of this genus display considerable reproductive diversity both between and within species [4]. Some Mexican cultivars of *V. planifolia* show natural self-pollination rates from 4–6% to 20% [5]. However, in Mexico, most varieties of this species, such as Mansa, are incapable of self-pollination [5] while some varieties are self-incompatible; for example, 80–100% of fruit of “Oreja de Burro” abort three months after self-pollination [6].

Plants attract their pollinators using sensory signals (flower shape, colour, fragrance), floral rewards (nectar, pollen, oils, resins), or deception (based on sex, food, brood-site) [7,8,9]. Within the orchid family, about 46% of the species display deception strategies to attract their pollinators, with food deception being the most common [10]. According to Lecomte and Chalot [11], Ridley [12], Bouriquet [13], Purseglove et al. [14], and Torregrossa [15], native pollinators of commercially grown species of *Vanilla* are rare and exist only in South and Central America. In 1836, Charles Morren reported that flowers of *Vanilla* spp. can only be pollinated by a tiny Mexican stingless bee of the genus *Melipona*. This bee does not occur elsewhere in the world where vanilla is grown commercially, and only 1% of flowers are naturally pollinated in Central America by the bee [16]. Bees of the genus *Tetragonula* (Meliponini) have been suspected of pollinating flowers of *Vanilla* spp. in Guadeloupe [17]. Dressler [18] believed that in the American tropics, a species of *Vanilla* is frequently pollinated by large bees of the genus *Eulaema* (Apidae: Euglossini) and doubted that a small bee of the Meliponini could be an effective pollinator.

Soto [5] reported the existence of three pollination systems among Mexican species of *Vanilla*. The first system, restricted to *V. indora* Schiede, involves carpenter bees of the genus *Xylocopa* (Apidae: Xylocopinae). The second, specific to *V. pompona* Schiede and *V. colombiana* Rolfe (cited as *V. hameri*), involves bees from the genus *Euglossa*, in which males are attracted to the fragrances produce by the flowers. The third system concerns *V. planifolia*, *V. odorata* C.Presl and *V. insignis* Ames that are pollinated through generalised food deception. The latter system is commonly observed in other orchid species that occur in low density populations [7]. Lubinsky et al. [19] conducted field observations of *V. planifolia* in Oaxaca in the spring of 2004. They noted occasional flower visits by ants and bees of the genera *Melipona*, *Euglossa*, and *Exeretes* but without the occurrence of pollination. In the Peruvian Amazon in September 2005, they observed that species of *Melipona* and *Euglossa* did not remove pollen from flowers of *V. grandiflora* Lindl. possibly because these insects are too small. However, successful pollination by *Eulaema meriana* Olivier was suspected following the observation of successful pollen removal by this orchid bee species [19]. Other studies have reported that flowers of *V*. *planifolia* were occasionally visited by hummingbirds [19,20]. Pansarin and Ferreira [21] observed a species of hummingbird (*Amazilia fimbriata* Gmelin) pollinating *V. palmarum* flowers, and Watteyn et al. [22] observed hummingbirds using their beaks to search for nectar in the flowers of *Vanilla hartii* Rolfe.

The floral structure of *V. planifolia* makes natural self-pollination difficult, as the rostellum prevents contact between stamens and stigmas. Some rare species of *Vanilla*, such as *V. palmarum* (Salzm. ex Lindl.) Lindl., *V. griffithii* Rchb.f., and *V. savannarum* Britton are self-pollinating [4]. *Vanilla planifolia* possesses a number of unusual features compared to the majority of orchids species. The emergent stigma (a membranous structure that projects well away from the body of the column) is three lobed, and the anthers and the median stigmatic lobes bend downward toward the lip of the flowers. The rostellum is part of the median stigma lobe and, if the flower could be insect pollinated, an insect would brush against this structure where it would be coated with glue and then pollinia or their caudicles. Flowers of *V. planifolia* bloom for only one day, opening early in the morning and must be hand-pollinated the same day before they close in the afternoon. Hand pollination is performed by lifting the rostellum back so that the pollen-bearing anther can be pressed against the stigma and accounts for about one third of the labour cost of growing vanilla. The ideal time for pollination is from 06:00 to 13:00, although the stigma remains receptive for up to 24 h [23,24]. Seed numbers per fruit are related to the number of pollen grains deposited on stigmatic surfaces during pollination [25].

To find ways of overcoming the costs associated with hand pollination, this study presents the outcomes of observations and experiments on the use of flies (*Lucilia cuprina* (Wiedemann) (Diptera: Calliphoridae)) and two native Australian stingless bees, *Tetragonula* (*Trigona*) *carbonaria* Smith (Apidae: Meliponini) and *Austroplebeia australis* Friese (Apidae: Meliponini) as pollinators, and the effects of these pollinators on fruit set, fruit size and yield of commercial vanilla plants grown in protective enclosures. *Lucilia cuprina* was chosen because calliphorid flies are effective pollinators of crops in enclosures or under nets [26]. The bees were chosen as Stehle [17] reported that bees of the genus *Tetragonula* have been suspected of pollinating vanilla flowers, and Watteyn et al. [22] observed that 69% of bee visitors approaching *V. hartii* flowers were a species of *Trigona*. European honeybees (*Apis mellifera* L.) were not tested as they can be aggressive in enclosed structures such as greenhouses. The main signals that flowers employ to attract their insect visitors are colour, scent and food-deception, and an analysis of all three was carried out to determine how vanilla flowers might advertise their themselves to potential insect pollinators or to recruit insects, such as ants, to protect the vanilla flowers. In addition, alternative hand pollination techniques were also examined.

## 2. Materials and Methods

### 2.1. Locations and Growing Conditions

We initially observed flowering in field-grown (forest) vanilla plants in the Daintree, where we recorded no floral visitors aside from ants, that were likely to be effective pollinators. Based on these observations, we selected several species of potential pollinators that occur naturally in Australia, including in Queensland, for further testing.

Three experiments were conducted in this study.

#### 2.1.1. Experiments 1 and 2—DVS Shade House

These were conducted at the property of Daintree Vanilla and Spices (DVS), Daintree Forest, Cow Bay, North Queensland (16.23° S, 145.45° E, and 116 m AMSL) during two flowering seasons in October (mid-spring). A closed shade house (11 m long × 8 m wide × 4 m high) was constructed on the property (Figure S1). The metal frame was clad with a green, woven, polypropylene net (Sarlon^TM^, Polyfab Australia Pty Ltd., Dandenong South, VIC, Australia) that transmitted 40–50% sunlight. The floor was covered with sand overlaid with a black, non-woven fabric (Weed Mat, Maccaferri Australia Pty Ltd., QLD, Australia) to prevent weed growth. The shade house was divided into two groups of four closed compartments separated by a central aisle with doors leading into each chamber. Five mature vanilla plants, grown in cut-down 20 L plastic drums filled with local tropical mulch were placed in each compartment (Figure S1); the various potential pollinating insects were confined within these compartments. No insecticides were applied during the flowering period. The plants were hand watered as required.

#### 2.1.2. Experiment 3—Controlled Environment Greenhouse WSU

This experiment was conducted at Western Sydney University, Hawkesbury Campus, Richmond, NSW (33.62° S, 150.75° E, and 20 m AMSL) from September to November in a sealed, controlled-temperature greenhouse compartment. The compartment was 5 m long × 3 m wide with a concrete floor. The greenhouse was of a metal frame construction and was double clad with 6 mm thick polycarbonate sheets (Quarantine Bioassay Insectary, Croudace Greenhouse International, Box Hill, NSW, Australia). Temperatures (minimum night 18 °C and average maximum day 28 °C) were maintained with a reverse cycle air conditioning unit (Carrier Air Conditioning Pty Ltd., Silverwater, NSW, Australia). Relative humidity (RH) was maintained automatically at 60–70% by misting (Seeley International Pty Ltd., St. Marys, SA, Australia). An internal shade screen (3 m above floor level) was opened or closed automatically, depending on the prevailing light intensity. Light intensities at midday in midsummer in the green house with the shade screen closed were about 15% of full sunlight. Five two-year old plants were grown in orchid potting mix (Brunnings, VIC, Australia) in each of two single flat beds that were 1.86 m long, 0.770 m wide and 0.160 m deep and lined with perforated black polyethylene sheeting. The beds were constructed of galvanised tubular steel and timber and were fitted with a trellis comprising tubular steel uprights at each end and a cross bar of polyvinyl chloride. Wooden stakes to support the vanilla plants were attached to the cross bar. The vanilla plants were hand watered as required and slow release fertiliser applied (Nitrophoska N:P:K 16:3:12.5 plus trace elements, Brunnings, VIC, Australia).

### 2.2. Insect Pollinators

#### 2.2.1. Experiment 1—DVS Shade House

For this preliminary experiment, a hive of the native stingless bee, *Austroplebeia australis*, was obtained from the Western Sydney University (WSU) apiary. The colony was managed in a half size Original Australian Trigona Hive (OATH) box [27] fitted with a 3 mm thick Perspex lid to enable observation. A wooden lid was attached to the top to exclude light. The colony was visually confirmed to be queenright and well populated with adults and was actively producing brood. This hive was placed in one compartment of the shade house. A colony of *Tetragonula carbonaria* in an OATH box was also obtained from the Western Sydney University apiary. The hive was confirmed to be queenright, full strength and well populated. This hive was placed in a second compartment. In both Experiments 1 and 2 (see below), to determine if any natural pollination took place and to act as a “no insect” control, the flowers in one compartment were left untouched and without added insects. The activities of the insect pollinators in the other compartments were observed daily from 08:00 in the morning until 13:00 in the afternoon when the flowers closed.

Three thousand sheep blowfly pupae (*Lucilia cuprina* (Wiedemann), (Diptera: Calliphoridae)) were purchased at the pupal stage from the Elizabeth Macarthur Agricultural Institute, Agriculture NSW, Sydney, Australia for Experiments 1 and 2; it took about four days for adult flies to emerge. This species of blowfly has been used successfully in Australia to pollinate a range of commercial vegetable seed production crops (Garry Levot, pers. comm.). The three thousand pupae were divided equally among three polypropylene containers (Genfac Plastics, Australia); one container was placed in each of three compartments. In addition, six racemes each of six plants were bagged with white mesh-net silk bags, and seven blowflies were enclosed in each bag to maximise the chances of blowflies visiting and pollinating the flowers (Figure S2).

#### 2.2.2. Experiment 2—DVS Shade House

Two hives of *T. carbonaria* were purchased from commercial stingless bee producers (Russell and Janine Zabel, Hatton Vale, Queensland) and taken to DVS. Each of the *T. carbonaria* colonies used in this trial was housed in an OATH box. While the hives were guaranteed to be queenright, it was not possible to check for the queen status of these colonies, as this practice is potentially damaging. Other non-invasive methods were employed to assess the health of the colonies. These included hive weight, audible entrance activity and forager flight activity. As such, the *T. carbonaria* colonies were considered at full strength. One hive was placed in each of two compartments in the shade house when 15–20% of the plants were flowering. The hives were placed on a small Table 450 mm height with the legs sitting in water to prevent ants from entering the hives.

#### 2.2.3. Experiment 3—Controlled Environment Greenhouse WSU

In Experiment 3, a colony of the stingless bee, *Tetragonula carbonaria*, in an OATH box was obtained from the Western Sydney University apiary. The hive was again confirmed to be queenright, full strength and well populated. This hive was placed in the controlled environment compartment on 10 October 2009.

### 2.3. Artificial Pollination

#### 2.3.1. DVS Shade House

Opened flowers on vines in two compartments of the greenhouse at DVS were hand-pollinated between 08:00–09:00. Hand-pollination was performed by holding the flower in one hand and pushing down on the labellum with the thumb to release the column (Figure S3A,B). The stamen cap was removed with a toothpick held in the other hand to expose the pollinia (Figure S3B). The toothpick was used to push the thin rostellum up under the stamen, and then the pollinia were brought into contact with the sticky stigma by pressing the stamen onto the stigma with the thumb and finger (Figure S3C). Finally, the column to which the pollen mass adhered was released (Figure S3D).

#### 2.3.2. Controlled Environment Greenhouse WSU

The vines flowered from early September until the end of October. Artificial hand pollination using the toothpick, as previously described and illustrated in Figure S3, and three alternative pollination techniques were performed twice a week on randomly selected flowers. Pollinated flowers were tagged with the date of pollination. The first two techniques involved collecting pollen from stamens cut from the flowers and applying it to the stigma of the same or different flowers with either a cotton bud or a small paint brush. For the third technique, fresh stamens were collected in the morning during flowering in November. The pollen was suspended in distilled water to which a few drops of the non-ionic surfactant, Tween 80^TM^ (Polysorbate 80, Sigma-Aldrich Pty. Ltd., Sydney, Australia), was added. The suspension was mixed thoroughly and sprayed onto the open flowers with a nasal atomizer. These treatments were applied in the morning between 9:00–11:00 a.m. during flowering in November.

The percentages of pod set were determined three days after each pollination treatment. All unopened flower buds were removed after eight to ten pods had set to optimise yield and pod size. Pod lengths and diameters were measured weekly for ten weeks, by which time bean growth had almost ceased.

### 2.4. Floral Attractants

Floral scent often plays a pivotal role in achieving successful pollination in many insect-pollinated plants. Therefore, volatiles released by flowers on vines grown in the greenhouse at WSU were analysed by gas chromatography-mass spectrometry (GC-MS), Varian Saturn 2000R (California, USA) and exudates released from glands at the base of the corollas were analysed by High-performance liquid chromatography (HPLC), Shimadzu LC-20A (Prominence, Japan). Flowers were also examined under A Rofin Polilight PL500 UV light (Rofin Australia Pty Ltd., VIC, Australia).

#### 2.4.1. Capture and Analysis of Volatiles Emitted by Vanilla Flowers

An unpollinated flower was enclosed in a glass test tube (190 mm long, 25 mm internal diameter) (Sigma, Australia) that was sealed with a screw cap containing a GC septum. Headspace volatiles were trapped on a solid phase microextraction (SPME) fibres (Supelco, Sigma Aldrich, Germany), Canon (Ōta, Japan) for 2 h at 20 °C. The fibres were inserted into the inlet of the GC and desorbed in the injection port for 10 min at 280 °C. The emitted volatiles were analysed using a Varian Saturn 2000R (California, USA) combined GC and MS in splitless mode. The system was fitted with a 1079 injector suitable for SPME analysis. This procedure was repeated ten times using freshly-harvested, unpollinated flowers and clean glass test tubes. No volatiles were released from the SPME fibres themselves. Identification of floral volatiles separated by GC-MS was carried out by comparing their mass with those in the NIST library 98, Wiley 8, that contains four data bases including MS library (National Institute of Standards and Technology, Gaithersburg, MD, USA). The results of the analyses are presented as relative abundances.

#### 2.4.2. Analysis of Nectar

Ten samples of nectar were collected each from a randomly selected flower from each of the ten plants in the chamber from the junction of flower buds and ovaries. Sample preparation for injection consisted of diluting the nectar in ultrapure water (1:5 (*v*:*v*)). Nectar samples were analysed using HPLC (Shimadzu LC-20A Prominence, Japan) fitted with a refractive index detector (Shimadzu RI-10A, Japan). Chromatographic separation was carried out using an Aminex HPX-87P column (300 × 7.8 mm; Bio-Rad, Sydney, Australia), and a 0.600 mL/min isocratic flow rate of 0.005 M sulfuric acid at 60 °C with a 10 μL injection volume. Sugars were identified by comparing the resulting chromatographs with those obtained from standard sugar mixtures. Separation of sugars occurred in 25 min chromatographic runs.

#### 2.4.3. Flower Colour

The reflectance of freshly harvested vanilla flowers was examined with UV light. A Rofin Polilight PL500 (Rofin Australia Pty Ltd., VIC, Australia), a specialised forensic light source that utilises a high intensity, 500 watt xenon arc lamp, was used to provide UV radiation. The UV filter in the camera provides UV radiation peaking at 350 nm with a bandwidth of 80 nm (UV-A). The irradiated flowers were photographed with a modified Canon EOS 30D (UV/IR barrier filter removed). Images were also taken using a Baader UV filter that only transmits radiation between 320–390 nm. The investigation was conducted in a dark room.

Photographs were captured with a Canon (Japan) 100 mm macro lens and a Canon MP-E65 macro lens that can produce up to 5× life-size magnification. The mirror was locked, and a remote shutter release mechanism was employed to further avoid camera shake. Exposure times varied between 5–30 s.

## 3. Results

### 3.1. Behaviour of Stingless Bees

#### 3.1.1. Experiments 1 and 2—DVS Shade House

The activities of *T. carbonaria* and *A. australis* were observed from the first day after the hives were placed in the greenhouse compartments at DVS. *Tetragonula carbonaria* was active (i.e., emerging from the hive entrance and flying) between ~09:00 and ~15:00. The number of bees exiting the hive was highest at the beginning of the experiment and declined during the investigation period. When the weather was suitable for flight, the bees continued normal hive maintenance activities, including removing debris or visited the flowers. Flight activity ceased with the development of heavy cloud cover and rain. The average visitation rate of *T. carbonaria* to the flowers was three bees per eight flowers. The bees approached the open flowers, hovered by them and then slowly landed on the leaves or the labellum. Initially, the bees chewed and scratched near the intact anthers, then slowly moved along the mid lobe callus into the base of the column. Each bee spent about 5–20 s in a flower and regularly moved backwards and forwards as if looking for food at the base of the labellum (Figure S4). After visiting a flower, the bees remained on the apex of the labellum for a few seconds and then exited the flower without touching any part of the stamens or the stigmas. During the trial, the bees were observed leaving the flowers without pollinia on any part of their bodies. Weather conditions at the time of pollinator visits were warm (25–28 °C) and sunny, with a gentle, fluctuating breeze. No bean pods developed following exposure to the bees, indicating ineffective pollination.

*Austroplebeia australis* seemed unwilling to work within the small shade house. The activity of the bees was limited, guards were monitoring the entrance, and they constructed a resin curtain to close the hive entrance and seal gaps in the lid (Figure S5).

#### 3.1.2. Experiment 3—Controlled Environment Greenhouse WSU

**Figure 1:**
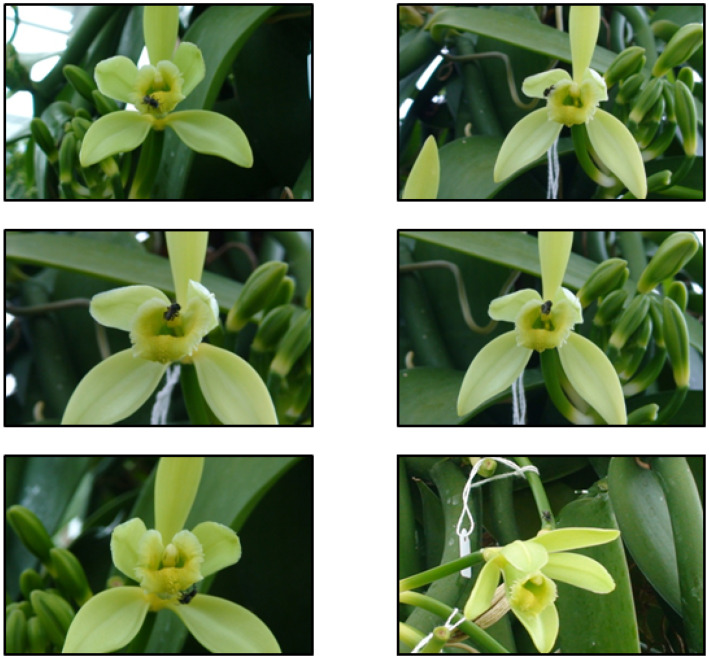
*Tetragonula carbonaria* collecting nectar from extrafloral nectaries of vanilla.

On the first day, the worker bees from the hive of *T. carbonaria* exited the hive and flew but landed on the walls and ceiling of the chamber. Many of these bees died. The vanilla flower has no internal nectaries, but there are three extrafloral glands at the base of the flower that continue to produce nectar during flowering and for about two months after pollination (Figure S6). Those bees exiting the hive on the second day quickly found the nectar produced by the extrafloral nectaries. Worker bees also alighted on the corolla and walked to the extrafloral nectaries at the base of the flowers but did not enter them (Figure 1). Unfertilised flowers closed in the afternoon the whole flower dropped from the raceme after 24–48 h.

### 3.2. Behaviour of Blowflies in the Shade House at DVS

Large populations of the blowflies emerged from their puparia within three to four days and initially walked on the ground until they commenced flying, after which they alighted on the netted walls of the shade house and commenced grooming themselves. After a long period (10–60 min) of resting or grooming, some of the blowflies took off and landed on the labellum of the flowers. When the flies entered the flowers, they travelled down the concavity of the labellum causing minimal labellum movement. The blowflies usually entered the flowers shortly after they were fully opened when temperatures were lower in the morning and remained in them most of the day (Figure S7). The number of flies in individual flowers varied considerably, particularly early in the investigation. Some flowers contained 6–12 flies while others received none; however, as the experiment progressed, the number of flies declined to approximately three per flower due to the number of surviving flies. Generally, the blowflies had a short life span of approximately six days in the shade house at DVS. Removal of pollinia from the stamens by the blowflies was not observed, but the flowers remained intact and, if pollination did not occur, the flower detached from the ovary and dropped off entirely within one to two days. Pollination was not successful even when flies were confined within bags around the flowers.

During the experiment in the shade house at DVS, the blowflies were attacked by green ants (weaver ants, *Oecophylla smaragdina* Fabricius) (Figure S8).

### 3.3. Artificial Pollination, Hand Pollination Using Toothpick or Alternative Pollination Techniques

Successful pollination in *V. planifolia* is denoted by the presence of drooping fruit (bean pods) on the racemes. The percentages of pods set and those dropping following hand-pollination using a toothpick or by transferring pollen to the stigma by brushing, using a cotton bud or spraying with a suspension of pollen were compared using a chi-square test. Highly significant differences were found among the treatments (χ^2^ = 154.0; d.f. = 3; *p* < 0.0001).

**Figure 2:**
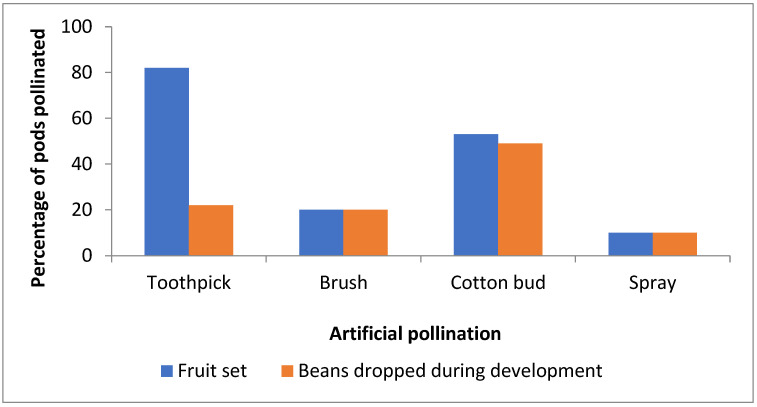
Percentage of bean pods set by different methods of artificial pollination.

**Figure 3:**
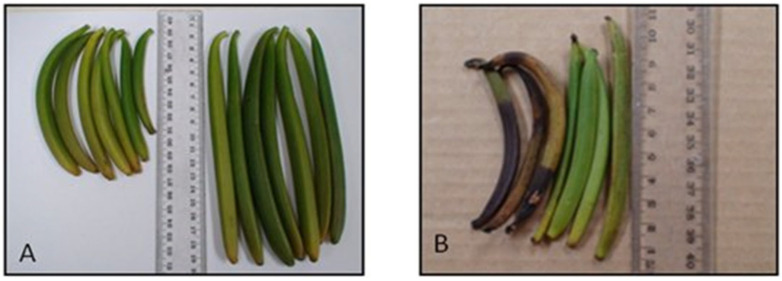
(**A**) Mature green pods from flowers pollinated with a cotton bud (**left**) showing their small size compared to those pollinated with a toothpick (**right**). (**B**) Some shedding of immature pods (**left**) occurred even when flowers are pollinated with a toothpick.

The traditional method of artificial hand pollination was the most successful, with over 80% of pollinated bean pods setting fruit compared to 10–53% for the other treatments (Figure 2). Also, highly significant (χ^2^ = 26.4; d.f. = 3; *p* < 0.0001) differences were found among the proportions of pods that were set but failed to mature and dropped from the raceme. Although the percentage of bean pods set by pollination with a cotton bud was higher than for the other two methods, the mature pods were not as long and were lighter than those pollinated with a toothpick and a high proportion of the pods dropped from the raceme (Figure 3A). Twenty per cent of pods that were pollinated with a toothpick also dropped during bean development on the vine; these were short and small (Figure 3B). Yellowing and fruit drop of immature fruits increased when temperatures exceeded 32 °C, when RH was below 80%, and during exposure for long periods to intense sunlight. Most fruit drop was noted within two months after pollination, mostly in December, at both the DVS and WSU sites.

### 3.4. Floral Attractants

#### 3.4.1. Nectar

The nectar predominantly comprised of sucrose (66%) and melezitose (30%) with nominal amounts of glucose and xylose as well as trace amounts of another unidentified monosaccharide (Figure S9).

#### 3.4.2. Floral Volatiles

The aroma of the vanilla flower could be detected by the human nose, smelling of freshly cut grass or being revolting, sour and metallic (SVD, pers. comm.); GC-MS detected 25 compounds. The compounds are grouped into classes which, to some degree, reflect their biosynthetic origin [28] (Table 1). They include monoterpenes, terpenes, sesquiterpenes, aromatics, nitrogen-bearing compounds and fatty acids related compounds. The most abundant chemical was the terpine, a-pinene (62.3%), followed by the monoterpene, eucalyptol (6.3%).

#### 3.4.3. Flower Colour

**Figure 4:**
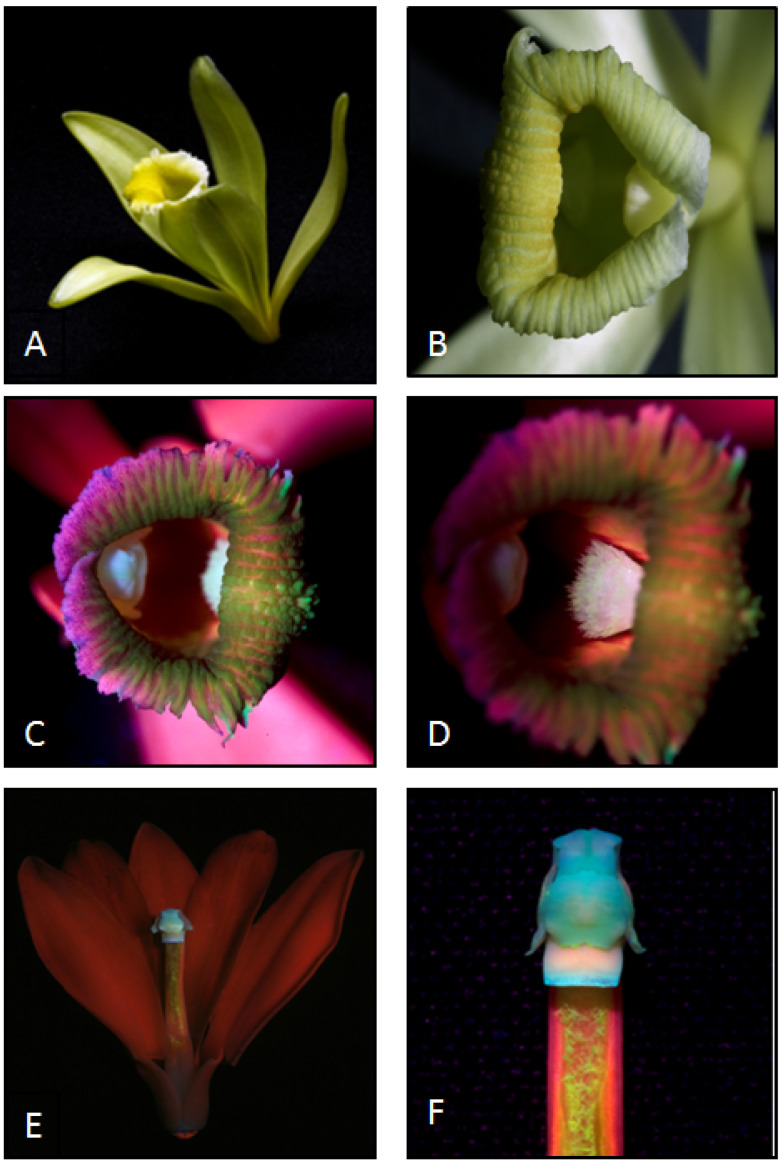
Vanilla flowers under white light (**A**,**B**). Under UV-A irradiation, the column and the callus attached inside the labellum fluoresce white (**C**,**D**), the petals fluoresce red and the stamens blue (**E**,**F**).

Flower colour is a primary attractant for insect pollinators and attraction is often triggered by colours emitted due to UV irradiation additional to colours visible to the human eye [29,30]. The flowers of vanilla are entirely pale green-yellow in visible light, but images of the flowers exposed to UV radiation revealed fluorescence from stamens, the column and the callus, which is located on the labellum (Figure 4).

## 4. Discussion

Orchids attract pollinators, using rewards, such as nectar, lipids, fragrances, pollen and resins, and various forms of deception [31,32], and many species of *Vanilla* are pollinated through food deception [21,33], through floral rewards of sugar-rich nectar, or by attraction via floral fragrances [34], or a combination of these [22]. Nectar production was not observed at DVS during the two flowering seasons but its production by the plants grown in the controlled temperature greenhouse at WSU was prolific. Both sets of plants were cultured from the same source, which suggests an environmental impact on the occurrence of nectar. The lack of apparent nectar production at DVS may be due to the glands not functioning; alternatively, the nectar may have been collected by the local insect population as quickly as it was produced, as the plants were exposed to the outside environment. The only insects observed on the plants were two species of ants that were found around the extrafloral nectaries. Lubinsky et al. [19] and Householder et al. [35] reported that ants are frequent visitors of flowers of *Vanilla* spp. but are not potential pollinators. The ants are attracted by the extrafloral nectar produced during bud development at the abscission layer between the bud and ovary [35,36]. Householder et al. [35] observed ants feeding on the sugary exudates of *V*. *cristatocallosa* Hoehne in Peru. The greenhouse used at WSU was insect-proof, and the plants were well watered, so ants were not a confounding factor for the pollination investigations.

The nectar produced by the plants of *V. planifolia* at WSU comprised predominantly of sucrose [22] and melezitose, with smaller amounts of glucose and xylose, plus a trace amount of an unidentified monosaccharide. Melezitose is produced in the honeydew of certain hemipterans and is highly attractive to ants [37,38]. Species of aphids that are tended by ants produce copious quantities of melezitose in their honeydew whilst the honeydew from those that are not tended lack this sugar [39]. The presence of melezitose produced from the extrafloral nectaries by *V. planifolia* may explain the concentration of ants around this organ and the apparent lack of production of nectar by the plants at DVS. In this study, the melezitose was formed in the elaboration of nectar by the plant and was not due to hemipteran insect activity [40] as the greenhouse was insect-proof and regular monitoring did not detect these insects.

*Tetragonula carbonaria* adapted to the environments in the shade house at DVS and the green house at WSU after several days, after initially landing on the roofs of these enclosures; this is a common occurrence (M. Duncan, pers. comm.). Subsequently, the bees visited the flowers and performed normal hive maintenance activities. Light and temperature are known to have a significant influence on the flight activity of *Tetragonula* [41]. In this study, the behaviour of the bees was influenced by light intensity and temperature. They did not leave the hives during cloudy weather, and the frequency of unfavourable weather conditions during flowering may have limited visits to vanilla flowers even if they could achieve effective pollination. The bees only foraged at temperatures of ~18 °C to just over 30 °C [SVD, pers. comm. and [42]], and 18 °C is considered to be the minimum at which this species will forage [41]. Ambient temperature conditions in the shade house were suitable for the activities of *T. carbonaria*; therefore, the lack of pollination is likely to be due to its size or behaviour. The small size of these bees enables them to easily enter and leave the flowers without touching the sticky stigmatic surface or inadvertently collecting pollinia. Lubinsky et al. [19] reported that no pollination occurred when flowers of *V. planifolia* and *V. grandiflora* were visited by bees of the genera *Melipona, Euglossa* and *Exeretes*; the authors suggested that this was due to their small size rendering them incapable of performing the required steps for pollination. Larger bees that can force their way past the sticky stigma and pollinia are, therefore, likely required. *Eulaema meriana* Olivier (Apidae: Euglossini), a large-bodied bee, was observed removing pollen of *V. grandiflora* in the Peruvian Amazon [19]. *Vanilla inodora* was visited by *Xylocopa* spp. [43], but no effective evidence of pollination by these carpenter bees has been presented [34]. The females of a *Xylocopa* sp. (Hymenoptera: Apidae: Xylocopinae) in the Serra So Japi reserve, São Paulo, Brazil, were observed perforating the base of the labellum searching for floral nectar produced by *V. edwallii* Hoehne, but never entering the tube formed by column and labellum.

There are larger *Tetragonula* species in other vanilla-growing regions of the world, and a *Tetragonula* sp. is suspected of pollinating vanilla flowers in Guadeloupe [17]. It is likely that in the American tropics, *Vanilla* spp. can be pollinated by large orchid bees of the genus, *Eulaema*, as suggested by Dressler [18], and pollinia of *Vanilla* spp. have been found on the bodies of such bees [44,45]. Orchid flowers are assumed to have adapted morphologically due to visits by pollinating insects. The ideal insect will have a thorax with a height and width that allows it to comfortably enter the flower but is also large enough to physically collect and remove pollinia [46].

Unlike *T*. *carbonaria*, at DVS, *A. australis* remained in their hive and blocked the entrance. The distribution of *A. australis* extends more than 2000 km, from the districts of Fitzroy and Central West Queensland, through the Darling Downs and into northern New South Wales [47]. The location of DVS lies north of this distribution. In contrast to *T. carbonaria*, which prefers areas of high rainfall and subtropical temperatures, *Austroplebeia* spp. thrive in areas that have more arid environments (A. Dollin, pers. comm.). A study by Halcroft et al. [47] on the nest environment of *A. australis* found a degree of thermoconformity and no evidence of brood cooling during the warmest months of the study. Greco et al. [48] reported that suitable conditions for *A. australis* to forage are light between 12,000–25,500 lx, temperatures of 21–26 °C and relative humidities between 42–55%. The temperatures at DVS were between 26–30 °C with relative humidities between 65–80%; light levels were not measured, but shade cloth provided 50% shade. Halcroft [49] reported that light intensity does not appear to affect the level of foraging activity in *A. australis* as much as temperature with ambient temperatures, as well as in-hive temperatures, having the greatest influence on the flight activity. Halcroft [49] predicted that climate change will have significant effects on the distribution of *A. australis*. Increasing ambient temperatures may see a gradual expanding of the southern distribution of *A. australis* and possibly other stingless bee species. Furthermore, there will be a 10% reduction in spring rain in southern Qld and northern NSW, thus increasing opportunities for foraging. Therefore, the environmental conditions experience by the bees at DVS may not have been conducive to their normal activities. However, had the environmental conditions been suitable, pollination is still unlikely to have occurred, due to the small size of these bees.

The adult blowflies were attracted to vanilla flowers, possibly because of their colour (pale green-yellow) [50] or for protection, but they failed to pollinate the plants. They also used the flowers as a place to rest, for shelter during windy and rainy weather, and possibly for thermoregulation, as the temperature in the flower tube may exceed the ambient temperature by up to 3 °C during the morning hours [51]. The flies contacted and entered the flowers more than the *Tetragonula* bees (see Figures S4 and S7) and also spent more time on or within a flower. However, the bees moved more frequently between flowers than the blowflies and were more active in flowers, apparently searching for rewards. During wet weather and when temperatures were lower, the blowflies spent long periods resting or grooming themselves on the column of the flower rather than feeding. Norris [52] reported that blowflies did not fly during wet, continuous rain. Flower visitations by blowflies can be to search for either pollen or nectar rich in carbohydrates to fuel flight and metabolic demands [53,54]. Our study found that green ants attacked and killed the blowflies. The green ant is used by some fruit farmers in the tropical north of Australia as well as in South-East Asia as a form of biological control to reduce the incidence of damaging insect pests [55]. The ant has territorial characteristics and was observed chasing away insects from flowers of *V. bahiana* Hoehne [33], and de Oliveira et al. [56] observed ants exhibiting floral herbivory on *V. planifolia* and *V. bahiana*. The presence of green ants may deter the activities of other potential insect pollinators, as these ants do not discriminate between helpful and harmful insects.

Vanilla flowers produce aroma volatiles that largely consist of α-pinene, eucalyptol, myrcene, silane, fluorotrimethyl 1, 2-benzenedicarboxylic acid, 3-carene, sabinene, limonene, linalool, and trans-caryophyllene; however, the impacts of these compounds on pollinator foraging are unknown. The responses of insect to chemical cues, including volatiles, varies greatly. For example, while eucalyptol can be used as an insecticide and insect repellent [57], it is one of many compounds that is attractive to males of various species of orchid bees who gather this chemical to synthesize pheromones [58,59]. In this study, the most abundant volatile was α-pinene (62% relative abundance). α-Pinene is a polyphenolic terpene and is a component of many aromatic plants, such as *Mentha* spp. [60]. It has been extensively studied for its relationships with insect behaviour and has been described as a major component of the floral scent of many plants [61] and has a variety of effects on insect behaviour. For example, Shahriari et al. [62] suggested that α-pinene can penetrate an insect’s respiratory system. For some plants, α-pinene may be related to protection against herbivorous insects; its production has been shown to increase in cotton when it is attacked by the beet armyworm (*Spodoptera exigua* Hübner) [63]. In addition, α-pinene reduces or masks the attractiveness of some plant volatiles to herbivorous insects [64,65], and is the most abundant compound (repellent) in the odour produced by male melon flowers [66,67].

Orchid flowers are a source of attractants for male euglossine bees, and approximately 650 orchid species are pollinated by these bees [68,69]. Many of the volatile compounds that these bees collect have been identified [70,71]. According to Soto-Arenas [5] and Dodson et al. [59], *V. pompona, V. hameri,* and *V. cribbiana* produce fragrances, such as limonene, attracting male euglossine bees, which is collected by them to attract mates. Interestingly, Dodson et al. [59] studied euglossine bees and suggested that α-pinene was not attractive for them. Similarly, Schiestl and Roubik [58] suggested that α-pinene could not be detected by the euglossine bees, *Euglossa cybelia* (Moure) and *Eulaema polychroma* (Friese). However, Blight et al. [72] showed that α-pinene could be used to initiate foraging in trained honeybees, but suggested that while it can be detected by honeybees, it may not be an attractant. Stökl et al. [73] found that α- and β-pinene and β-myrcene are key components of the oviposition site mimicry employed by the terrestrial orchid, *Epipactis veratrifolia* Boiss. & Hohen., which mimics the volatile compounds of aphids to attract and induce oviposition behaviour in several species of female, adult hoverflies as pollinators; the resulting larvae then potentially act as aphid predators.

The odour profile of vanilla flowers is remarkably similar to the alarm pheromone released by several aphid species, such as *Megoura viciae* Buckton. In our study, α-pinene is likely to be an attractant to stingless bees, and limonene and the aromatic compounds emitted by *V. planifolia* may also be attractants. In field bioassays, linalool produced by several perfume-rewarding orchids has also been shown to be an attractant to euglossine bees [70,74]. The cyclic sesquiterpene, trans-caryophyllene, identified in this study (albeit at a low level of 0.4%) has been found to be an attractant or a component of an attractive blend in several rewarding orchid species [75], but is more commonly associated with herbivory and pathogen resistance than pollination [76]. The potential role of the floral fragrances we have identified in *V. planifolia* should be further investigated to determine whether they are repellents or attractants.

In relation to flower colour, at DVS where the vanilla vines were grown in a shade house in natural light, the *Tetragonula* bees approached the flowers, touched them, and then alighted in an upright position with their heads directed toward the top of the column, which is the most fluorescent organ under UV radiation. However, the behaviour of the *Tetragonula* bees in the polycarbonate green house at WSU was different. The bees emerged from their hive, circled and flew to the greenhouse roof and only a few returned to their hives. After a few minutes, some bees flew as if they were going to land on the flowers but once they were close to them, they landed near the extra floral nectaries. Absorption of UV radiation by the polycarbonate cladding of the greenhouse may explain the different behaviour of the bees at WSU. Kwon et al. [77] reported that polycarbonate sheets entirely blocked radiation in both the UV-B (300–320 nm) and UV-A (320–400 nm) ranges. Morandin et al. [78] showed that in a greenhouse clad with plastic foil, bee activity was higher and the losses from colonies were lower when the cladding transmitted ultraviolet radiation, rather than when it absorbed most of the UV radiation.

Blowflies are able to distinguish colour and can discriminate between yellow and blue but prefer yellow, except when decaying protein odour is present when brown or purple is preferred [79]. Some flowers without these brown or purple colours have evolved carrion-like odours that attract flies [80], while others present features that look like images towards to which the flies are attracted. In general, flies are attracted to blossoms with regular and simple shapes, to pale colours [81], to the presence of nectar resources, and to the production of a suitable odour. The nectar must be readily available to the flies, and the plant’s sexual organs should be exposed for pollen to be transferred proficiently. Flower visitations by blowflies can be to search for either pollen or nectar rich in carbohydrates [54] and may inadvertently cause cross pollination [82]. In this study, it appears that the blowflies were attracted to the yellow-green vanilla flowers and were possibly searching for nectar. However, it is unlikely that the odour emitted by the flowers is an attractant, as a-pinene is repellent to the house fly, *Musca domestica* L. [83]. No pollination resulted from the visits of the blowflies.

The traditional method of hand pollination of *V. planifolia* was developed in 1841 on the island of Reunion by Edmond Albius, a young slave [84], and personnel trained in this technique can pollinate 1000–2000 flowers per day [14]. This technique is laborious, requires fine motor skills [85] and can account for 40% of the production costs [86]. Gregory et al. [86] used various techniques to apply the auxins, IAA, IBA, 2,4-D and NAA, as well as gibberellin to flowers to stimulate parthenocarpic fruit production. The auxins stimulated production; however, the fruit were of smaller than those that were set using the traditional toothpick method. The chemical composition of the pods was not examined. In the current study, three mechanical methods of pollination were trialled, but each resulted in lower pod set than the traditional method with the bean pods being smaller in size and with a greater number dropping from the raceme. These results support the contention by Lescourret et al. [87] that a high number of pollen grains needs to be transferred to achieve higher seed numbers and maximum fruit size.

## 5. Conclusions

This study concluded that the Australian stingless bees, *T. carbonaria* and *A. australis*, are too small to remove the pollinia and pollinate the flowers of *V. planifolia*. Although blowflies regularly visited the flowers, they also did not affect pollination. In addition, variations on the traditional method of hand pollination using a toothpick were trialled; however, compared to the traditional method, all resulted in lower pod set and the production of inferior quality fruit. Further research is required to determine if there is a native Australian insect that can effectively pollinate vanilla and that can also be managed in an enclosed greenhouse production system. Bumble bees (*Bombus* spp.; Hymenoptera: Apidae) are large, hairy species, mainly endemic to the cool climates of the northern hemisphere but possibly might pollinate vanilla flowers in controlled temperature greenhouses; however, the introduction of bumble bees may possibly have negative impacts on native flora and fauna [88]. Large carpenter bees (*Xylocopa* spp.) are common visitors to flowering plants in the tropics and subtropics [89] and can tolerate extremes of temperature and variable degree-day heat accumulation. Furthermore, they can perform pollination under conditions of rainfall and heavy winds. These traits make them attractive candidates for agricultural pollination in hot climates, particularly in greenhouses [90]. Carpenter bees have been demonstrated to be efficient pollination of passionflower [91], blueberries and greenhouse tomatoes in Australia [92] and greenhouse melons [93]. Blue-banded bees (*Amegilla* spp.) play an important role in the pollination of cucurbitaceous and solanaceous plants [94] and are potential pollinators of greenhouse tomatoes [95]. Tomato yield was increased in greenhouses due to the pollination activity of *Amegilla chlorocyanea* Cockerell [96]. Therefore, both blue-banded and carpenter bees have the potential to pollinate *V. planifolia* in controlled environment greenhouses and could be investigated. However, these bees are solitary rather than eusocial (living in hives), so would require development of management plans in protected cropping scenarios such as vanilla. Furthermore, additional investigations should be conducted in a range of open-field vanilla cultivations to identify any floral visitors, and to subsequently evaluate their effectiveness as pollinators within controlled, closed environments.

## Figures and Tables

**Table 1:** Chemical composition of volatiles produced by vanilla flowers (RI = retention index). The compounds were identified with reference to the NIST98, MS library and Wiley 8.

Compounds	RI	Relative Abundance (%)
**Monoterpenes**		
3-carene	0903	0.9
sabinene	1028	0.4
myrcene	1076	4.6
eucalyptol	1217	6.3
ocimene	1225	3.1
alloocimene	1504	0.5
geranyl acetone	2391	0.5
myo-X	1540	0.5
**Terpenes**		
limonene	1204	0.6
citronellene	2632	0.3
α-pinene	1259	62.3
linalool	1423	0.6
verbenene	15.17	0.3
myrtanol acetate	1715	0.5
**Sesquiterpenes**		
trans-caryophyllene	2786	0.4
**Aromatics**		
benzaldehyde	1004	1.7
1, 2-benzenedicarboxylic acid	2733	3.7
benzeneacetaldehyde	3026	0.4
N-hexyl salicylate	3208	1.5
**Nitrogen containing compounds**		
dimethylamine	0140	1.4
**Fatty acids related compounds**		
1-dodecanol	1607	0.6
undecanal	1439	0.7
**Other compounds**		
silane, fluorotrimethyl	0335	3.9
benzophenone	2839	2.8
4-heptanol	2769	1.5

## Data Availability

Data will be made available on request.

## Supplementary Materials

The following supporting information can be downloaded at: https://www.mdpi.com/article/10.3390/plants13212977/s1, Figure S1: Shade house at DVS. (A) Floor plan of the shade house; (B) the potted vines placed within a compartment; Figure S2: Blowflies within a silk bag enclosing a raceme with an open flower; Figure S3: Hand pollination of *V. planifolia* flowers. Figure S4. *Tetragonula carbonaria* bees foraging on vanilla flowers. Figure S5. Entrance to the hive of *A. australis* closed with a curtain of resin Figure S6. Extrafloral nectaries located at the junction of flower buds and ovaries and on the blossom ends of developing fruit about two months after pollination. Figure S7. Vanilla flowers visited by blowflies (A–C); (D) non-pollinated raceme showing ovaries (the ovaries without an attached shrivelled corolla). Figure S8. Green ants attacking *Lucilia cuprina*. Figure S9. Chromatogram showing the composition of nectar produced by vanilla flowers.
