# Supplementary material for: Vanilla planifolia: Artificial and Insect Pollination, Floral Guides and Volatiles
Source: Plants (Basel). 2024 Oct 25;13(21):2977. doi: 10.3390/plants13212977 (PMC11547731; doi:10.3390/plants13212977)
Supplement: Supplementary file 1 [file plants-13-02977-s001.zip › plants-3228627-supplementary.pdf]

Supplementary Figures:

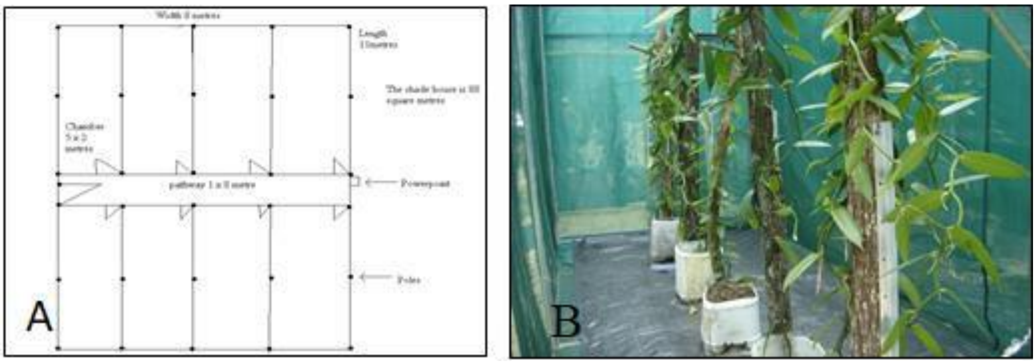

Fig. S1. Shade house at DVS. (A) Floor plan of the shade house; (B) the potted vines placed within a compartment.

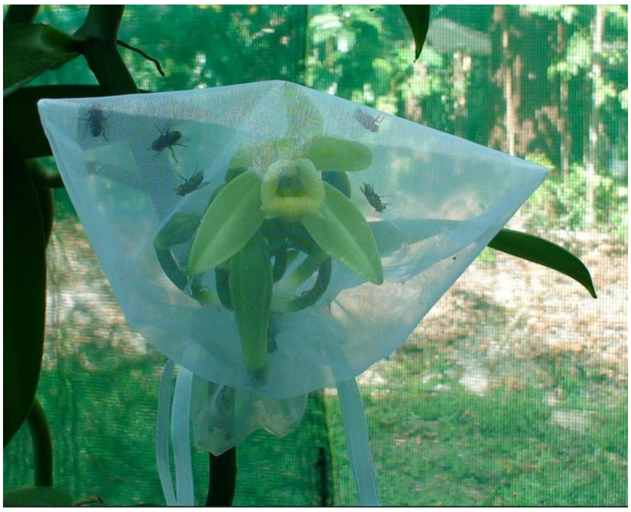

Fig. S2. Blowflies within a silk bag enclosing a raceme with an open flower.

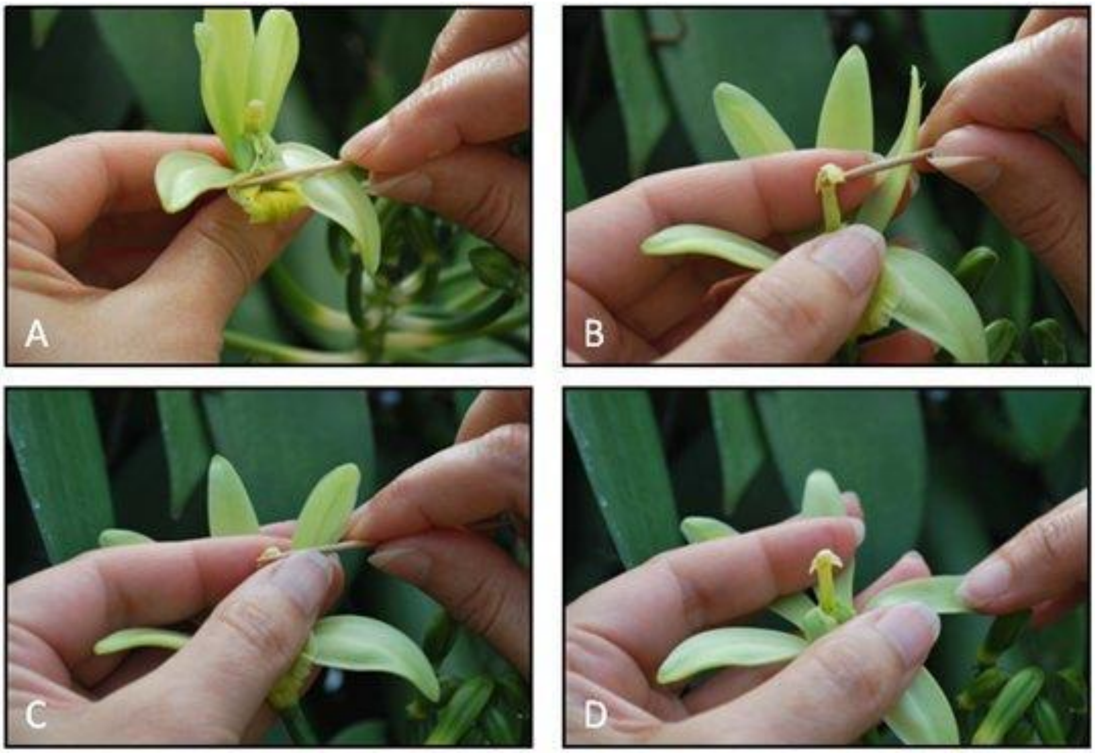

Fig. S3. Hand pollination of *V. planifolia* flowers. The labellum is pushed down to release the column (A), the rostellum is then pushed up under the stamen (B), the thumb and finger are used to press the pollinia against the sticky stigma (C), the column is released after pollination has been completed (D).

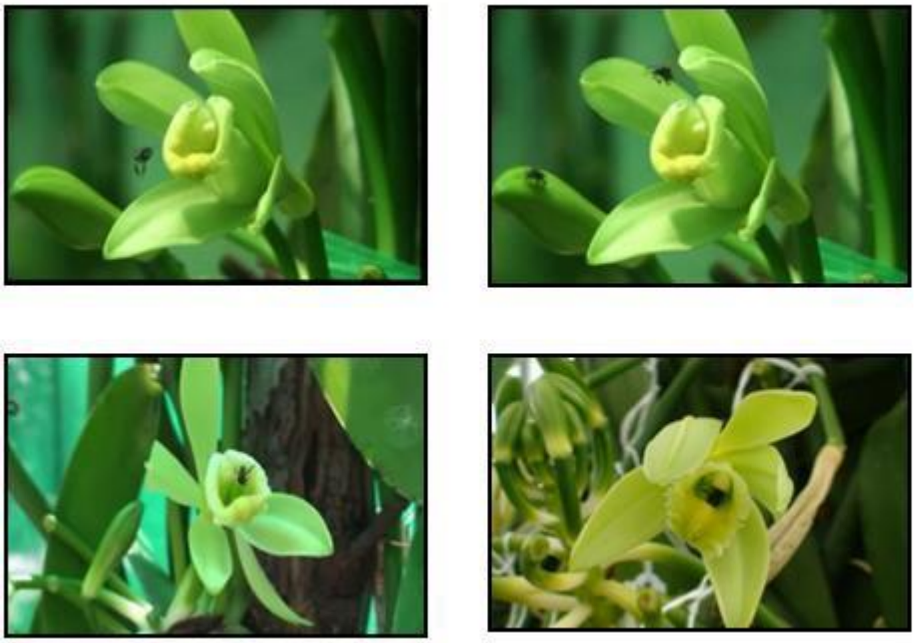

Fig. S4. *Tetragonula carbonaria* bees foraging on vanilla flowers.

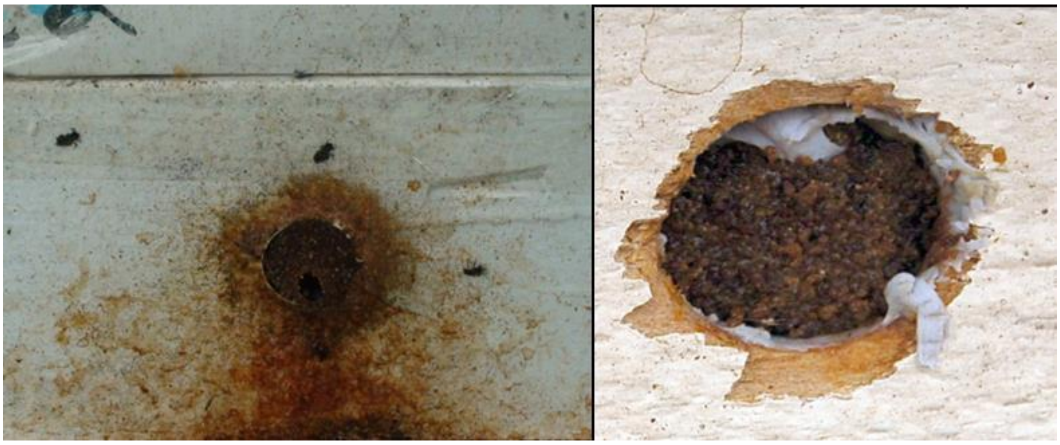

Fig. S5. Entrance to the hive of *A. australis* closed with a curtain of resin.

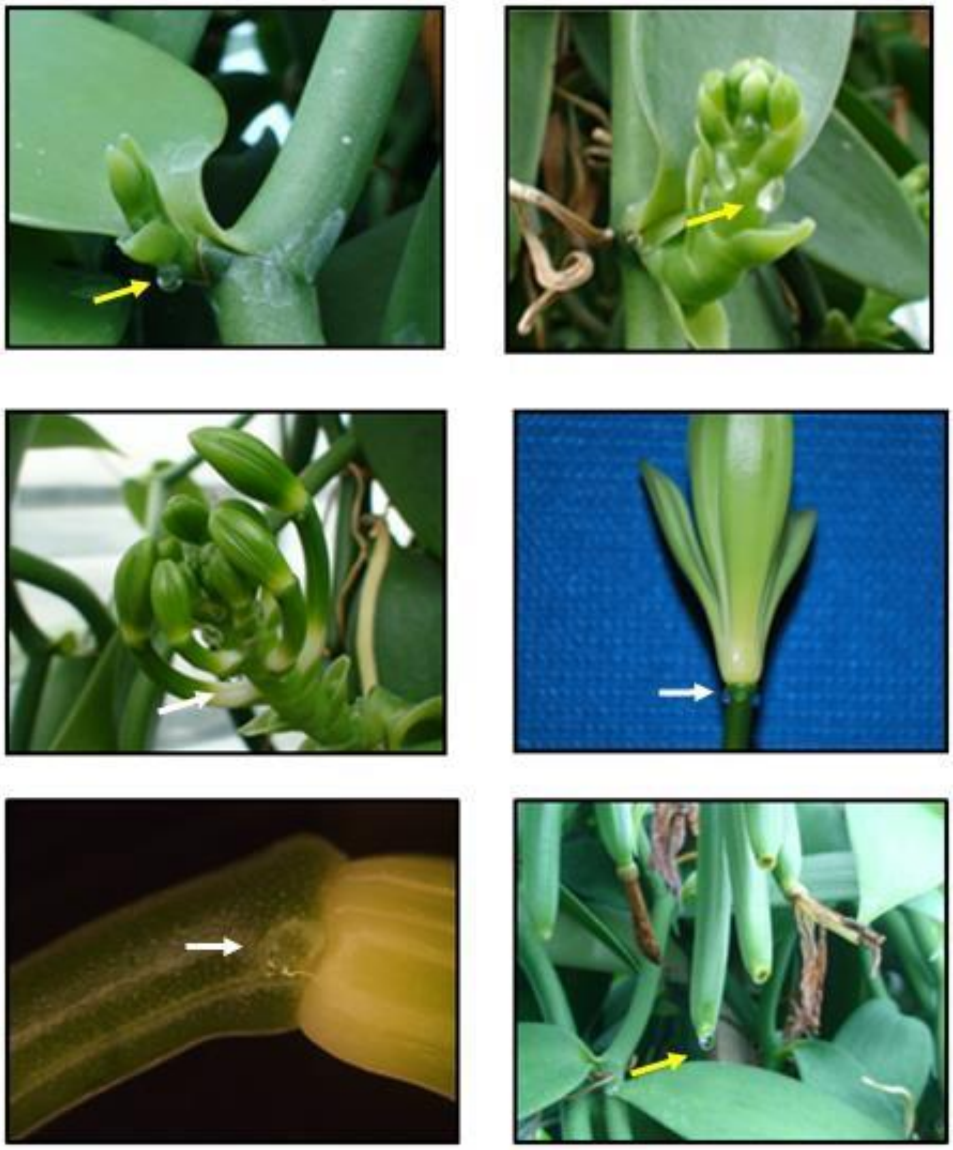

Fig. S6. Extrafloral nectaries located at the junction of flower buds and ovaries and on the blossom ends of developing fruit about two months after pollination. White arrows mark the positions of extrafloral nectaries, yellow arrows mark the production of extrafloral nectar.

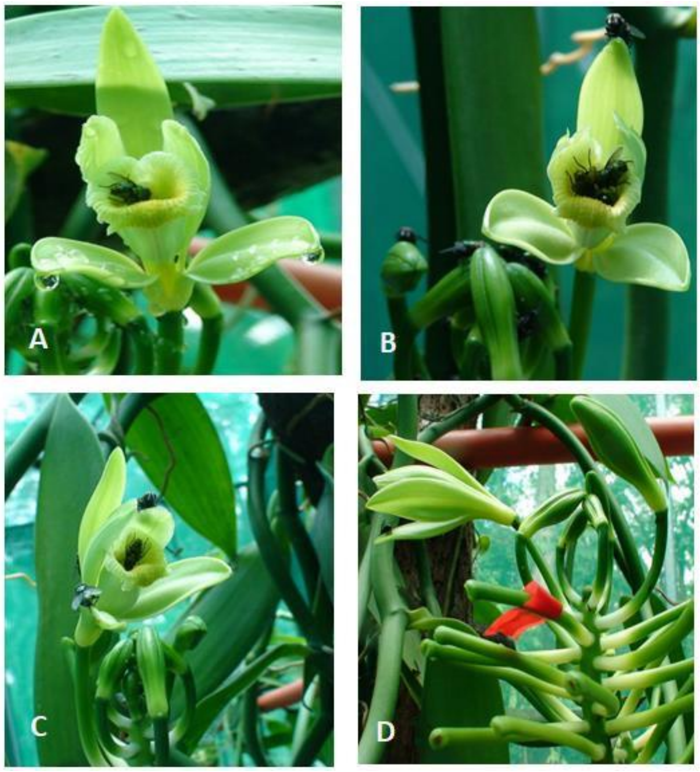

Fig. S7. Vanilla flowers visited by blowflies (A-C); (D) non-pollinated raceme showing ovaries (the ovaries without an attached shrivelled corolla).

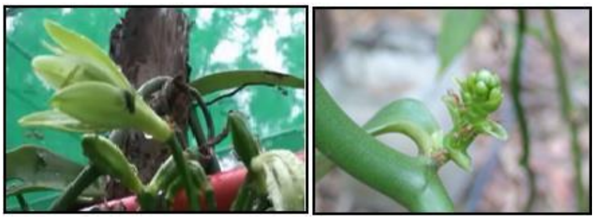

Fig. S8. Green ants attacking *Lucilia cuprina*.

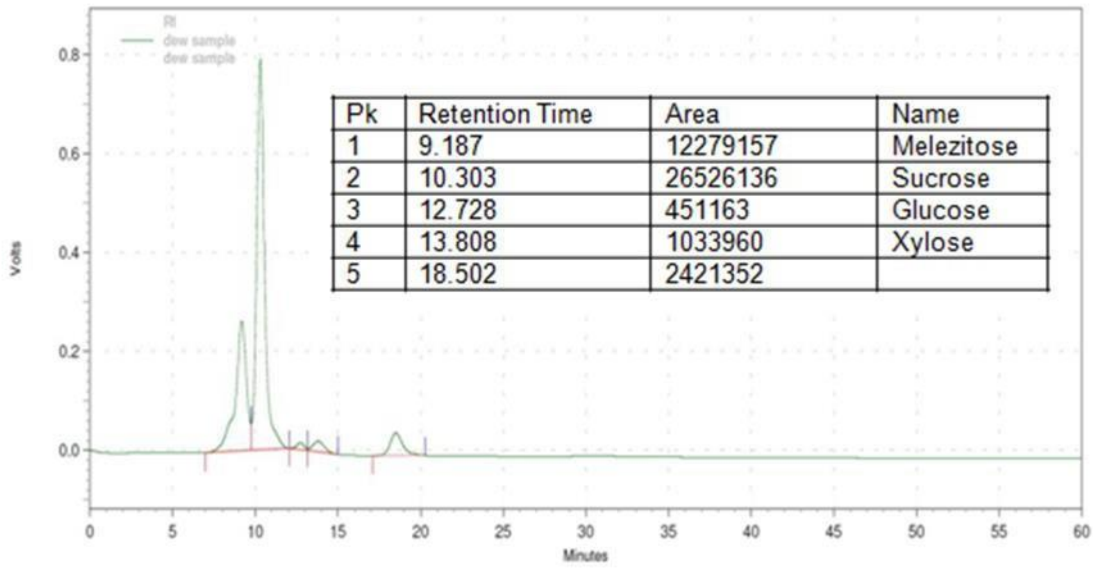

Pk	Retention Time	Area	Name
1	9.187	12279157	Melezitose
2	10.303	26526136	Sucrose
3	12.728	451163	Glucose
4	13.808	1033960	Xylose
5	18.502	2421352	

Fig. S9. Chromatogram showing the composition of nectar produced by vanilla flowers.
